# Long‐term smell loss experiences after COVID‐19: A qualitative study

**DOI:** 10.1111/hex.14018

**Published:** 2024-03-18

**Authors:** Hafize Özdemir Alkanat, Selda Arslan

**Affiliations:** ^1^ Department of Internal Medicine Nursing, Faculty of Health Sciences Giresun University Giresun Türkiye; ^2^ Department of Internal Medicine Nursing, Nursing Faculty Necmettin Erbakan University Konya Türkiye

**Keywords:** COVID‐19, long term, qualitative, smell loss

## Abstract

**Objectives:**

Sudden smell loss is one of the early symptoms of COVID‐19. Although it is stated that the loss of smell and taste following COVID‐19 improves within a few weeks, there are also cases that do not improve for a long time. The aim of this study is to reveal long‐term smell loss experiences after COVID‐19.

**Methods:**

A qualitative approach was adopted. We conducted semistructured interviews with 11 participants who had smell loss for at least 3 months. Interviews were recorded, transcribed and evaluated using a thematic analysis for qualitative data.

**Results:**

Nutrition and appetite, personal hygiene, threats to safety and emotional changes were the main themes created by the authors and were the areas where participant expressions focused. The participants used oral/nasal corticosteroid therapy for smell loss and received short‐term olfactory training, but could not find a solution.

**Conclusions:**

Long‐term smell loss problems, which were neglected during the pandemic period, should be carefully evaluated due to their negative effects. Understanding and focusing on the negative effects of loss of smell may contribute to the solution of long‐term smell loss problems.

**Patient and Public Contribution:**

Eleven participants who experienced long‐term loss of smell following COVID‐19 contributed to the study. They enriched the study by describing the effects of their experiences. There was no other participation or contribution from the public to the research.

## 1 INTRODUCTION

The World Health Organization declared COVID‐19 a pandemic, and this is the first pandemic in history caused by coronaviruses. The symptoms of COVID‐19 typically include dry cough, fever and dyspnoea.^1^, ^2^, ^3^ As a sudden inability to smell (anosmia) became the first reported symptom in countries with the most cases, it was accepted as an early symptom of COVID‐19.^4^, ^5^, ^6^ Smell loss arising due to COVID‐19 is described in the literature by the term ‘post viral anosmia’ associated with neuroinflammation.^7^, ^8^ Individuals who developed COVID‐19 usually did not recognize mild anosmia, which can be detected by an objective self‐evaluation.^9^ It is clear that the prevalence would be even higher by an objective self‐evaluation instead of patient self‐report. According to the major findings reported in a meta‐analysis, the prevalence of anosmia, at 53.5%, was the second main symptom after fever (62.2%).^10^ In another meta‐analysis, it was reported that 47% of 3563 people had taste and smell loss, of which 31% was severe and 67% was moderate–mild.^11^ Some patients wait for their ability to smell to come back on its own, but others seek medical attention including corticosteroid therapy or olfactory training, which has shown promising results in improving olfactory function.^12^ The recovery period of smell and taste loss has been reported to be 3 weeks (7 days on average).^13^ In certain cases, the healing process may take longer.

Long‐term loss of smell can result in a number of issues since the sense of smell is an essential alarm system that regulates food intake and plays a role in social relationships. The negative effects of loss of smell include decreased pleasure in eating, loss of appetite, difficulty in cooking, inability to recognize spoiled food, change in body weight,^14^, ^15^, ^16^ decreased safety,^14^, ^17^, ^18^, ^19^, ^20^ concerns about personal hygiene,^21^ feelings of vulnerability, moods swings and depression and a decrease in social interactions,^22^ professional success and sexual health.^16^, ^20^, ^21^, ^22^, ^23^, ^24^ Patients who have lost their sense of smell express difficulties with daily tasks and a decline in their quality of life.^23^ While there are studies on the prevalence of ^10^, ^11^ and treatment ^6^, ^7^, ^25^ for smell loss related to COVID‐19, there are not many qualitative studies that have analyzed in depth how patients are affected by long‐term loss of smell.

The aim of this study is to report long‐term smell loss experiences of patients after COVID‐19. The results of this study can be utilized to improve participants' outcomes by enabling us to identify patients who are experiencing smell loss, identify their needs based on their experiences and develop therapies accordingly.

## 2 MATERIALS AND METHODS

### 2.1 Design and sample

The research is a qualitative study using semistructured interviews conducted using a descriptive phenomenological design and thematic analysis. The qualitative methodology can provide straight descriptions of phenomena and provide answers to questions as to whom, what and where.^26^

The purposive sampling method was used in the study. The sample included individuals who met the inclusion criteria and agreed to participate in the study. The criteria for inclusion in the study were as follows: (1) individuals who experienced loss of smell in the last 3 months due to COVID‐19 and (2) individuals between 18 and 64 years of age. Individuals experiencing loss/disorder of smell for another reason (sinus diseases, neurological diseases, etc.) were excluded from the study.

### 2.2 Data collection

People who stated that they had lost their sense of smell for at least 3 months following COVID‐19 were recruited into the research via an online advertisement (Facebook 345.0.0.34.118 Meta Platforms Inc., WhatsApp Messenger 2.21.25.11 WhatsApp LLC). The sample size was determined according to data saturation, that is, the point at which no new themes emerged from the participants' experiences.^27^ The interviews were planned according to the preferences of the participants. Moderation was done by the first author (H. Ö. A.) and co‐moderation was done by the author (S. A.), who has both experience and training in conducting qualitative research. The interviews were conducted by both researchers.

Before the meeting, both informed consent and permission to record the interviews were obtained from the participants. Data were collected between August and December 2021. Descriptive characteristics and a semistructured interview form were used in the study. The semistructured interview form consists of three questions:

1. What difficulties have you experienced due to loss of smell after COVID‐19?
2. How did the loss of smell affect your daily life (physical, emotional, social and others)?
3. What methods have you used to deal with smell loss? (consulting a doctor, seeking medical and complementary treatment).

Two investigators participated in online Zoom meetings (Zoom Video Communications Inc. [version 2020]) with 11 participants, each lasting 30–45 (39.0 ± 4.97) min. During the interviews, the positive and negative experiences due to the COVID‐19‐related loss of smell were discussed. There were no refusals or dropouts among the participants. Notes were taken and audio‐recorded during the interview. The interview continued until the participants indicated that they had expressed themselves adequately and data saturation was achieved.

### 2.3 Data analysis

The data were analyzed using the seven‐step Colaizzi method, which is appropriate for descriptive phenomenological analysis.^28^ The following steps were involved in the analysis of the data: (1) The sound recordings were transcribed verbatim in order to obtain a written database. The transcribed data were read carefully several times to make sense of them as a whole. The data obtained from each participant were coded as P1, P2,…, P11 to ensure anonymity. (2) The sentences relevant to the phenomenon under investigation were noted down next to the interview documents. (3) Then, the data were classified and codes were created. The researchers listed the codes that they created. (4) The codes were then arranged in meaningful clusters based on their similarities. Themes that represent wider categories of information and that are used to explain the key findings of the study were obtained from similar codes. (5) The results were compiled in the form of a comprehensive description. (6) Participants' responses about their experiences and perceptions for each topic were condensed. (7) In the final step, feedback was obtained from two randomly selected participants to evaluate the themes obtained in the study. Both researchers conducted data analysis independently. Then, the researchers conferred together several times to discuss and compare their notes until they agreed on the themes. The data were handled and analyzed manually.

The reliability of the findings was enhanced by the authors consulting with each other and returning to the original transcripts. Two co‐authors analyzed the transcripts independently by bracketing data on preconceived ideas and strictly following the adapted Colaizzi's method described above. Findings were then compared and discussed by the team until consensus on themes, theme clusters and categories was achieved. Transferability was established by considering variations of participant characteristics and sufficient quotations collected through in‐depth interviews.^28^

### 2.4 Ethical considerations

Ethical approval (2021/12‐60 number) was obtained from the University Clinical Research Ethical Committee and the Ministry of Health to carry out the study. The Consolidated Criteria for Reporting Qualitative Research (COREQ) checklist was used to report the study.^29^ Written informed consent was obtained from all participants.

## 3 RESULTS

A total of 11 participants, 7 females and 4 males, aged between 18 and 56 years, participated in our study. It was determined that the participants' loss of smell lasted for 3–12 months after COVID‐19 and that the participants did not have a history of smoking (Table 1). All the participants were Caucasian. It was found that participants usually experienced loss of smell in the first week of the COVID‐19 infection; loss of smell and taste was the first symptom that led the participants to undergo a PCR test. All participants in our study reported that they received COVID‐19 treatment at home and were not hospitalized.

**Table 1:** Demographics and clinical characteristics of participants.

Participant (P)	Age	Gender	Marital status	Working status	Chronic disease	Smoking	Duration of Smell loss, months	Taste loss
P1	39	F	Married	Housewife	No	No	5	Yes
P2	42	F	Married	Officer	No	No	8	Yes
P3	38	F	Married	Academician	Migraine	No	3	Yes
P4	28	F	Married	Teacher	No	No	5	Yes
P5	49	F	Married	Academician	Diabetes	No	12	Yes
P6	42	F	Married	Nurse	No	No	11	Yes
P7	36	M	Married	Technician	No	No	9	Yes
P8	42	M	Married	Employee	No	No	6	Yes
P9	56	F	Married	Housewife	Hypertension	No	8	Yes
P10	18	M	Single	Student	No	No	9	Yes
P11	40	M	Married	Academician	No	No	9	Yes

Abbreviations: F, female; M, male.

Based on semistructured interview forms, 17 subthemes and 5 main themes emerged. The themes are ‘Nutrition and Appetite’, ‘Personal Hygiene’, ‘Safety Threat’ and ‘Emotional Reactions’ and ‘Coping with Smell Loss’. Codes, subthemes and themes are presented in Table 2. The individual quotes presented under each theme are examples of the challenges that participants often described. These quotes were selected from the striking nuances of participant descriptions that enabled the creation of subthemes. After the participants' statements, participants' number, gender (F/M), age and duration of smell loss (months) are given in parentheses.

**Table 2:** Codes, subthemes and themes.

Codes	Subthemes	Themes
Inability to taste, metallic flavour, excessive use of salt, sugar and spices, inability to enjoy meals, avoidance of high‐calorie foods and weight loss are all symptoms of terrible taste.	Diet changes	Nutrition and appetite
	Weight changes	
	Appetite changes	
	Search for flavour	
A bad odour, often changing clothing, using perfume or deodorant frequently, regular washing, a change in perspiration scent, more self‐care, questioning family members about your personal odour, disliking your body and a change in self‐esteem.	Taking shower often	Personal hygiene
	Using perfume/deodorant	
	Changing clothes	
Burning food, eating damaged food, being insensitive to noxious scents like smoke or gas and being afraid of being sick.	Prone to accident in the workplace	Safety threat
	Burning/poisoning risk	
Lack of sense of smell (house, flower, children) for a long time, lack of social ties, lack of social bonds, missing the scent of their loved ones, nature, and home.	Longing	Emotional responses
	Desperation	
	Social isolation	
	Anxiety	
	Depression	
	Altered body image	
Smelling rose, thyme, eucalyptus and lavender oil, using steroid nasal drops, looking for remedies on social media, seeing a doctor and failing to find a cure despite efforts.	Seeking medical help	Coping with smell loss
	Applying integrative therapy	

### 3.1 Nutrition and appetite

This theme explores how participants describe their experiences of changes in their appetite, eating habits and, in turn, their weight as a result of loss of smell/taste. Participants in the study reported experiencing loss of taste, metallic taste, increased use of salt, sugar and spices, inability to enjoy food, avoidance of high‐calorie foods and weight change due to loss of smell and taste.

Because I couldn't taste anything, I started to add more salt to my food and consume spices frequently. 4‐5 months after diagnosis, I had the smell and taste of blood in my mouth and a strange taste that I can only describe as baby powder. (P2, F, 42, 8m)

Taste loss came back but my sense of smell didn't come back right away. Then my sense of smell came back, oh how I wish it didn't. Now I smell stinky stuff like burnt tire and carrion. (P4, F, 28, 5m)

I have given up eating it because I can't smell or taste it; for example, I love hazelnuts, but I don't eat more of anything so as not to consume calories. (P5, F, 49, 12m)

I can distinguish sour, salty and sweet stuff but I can't taste the flavour of things I eat. I started to taste something different and bad while eating meat, chicken, fish, eggs, onion and garlic. Something spoilt like the smell of carrion, which bothered me. That's why I can't go to dining hall and I can't go out for dinner with my friends. I can no longer eat meat; if I force myself to eat, I vomit. I had stomach surgery before and lost weight, but for months I have been eating non‐nutritious foods such as yogurt, pasta and potatoes, and I have started to gain weight again because of junk food. (P6, F, 42, 11m)

I like drinking milk but now even if I drink low‐fat milk, it tastes intensively fatty and smells strong. Even if the chocolate I eat is not bitter, it tastes bitter. I have difficulty eating oily foods such as eggs, butter and fried food. As a consequence, I lost 4‐5 kg but I am not complaining about it. (P10, M, 18, 9m)

### 3.2 Personal hygiene

This theme explores how participants' hygiene habits in their daily lives changed due to loss of smell. Participants reported that after experiencing smell loss, they changed clothes frequently, used perfume or deodorant frequently, showered regularly and felt the need for more personal care when they perceived a bad smell emanating from their body. They stated that they tended to ask their family members about personal odour and experienced a change in self‐confidence due to discomfort in smell change.

Even my sweat and feces smell strange. I can't describe this smell, I smell bad scents that I have never smelled before in my life and I am worried other people can smell them too. I even miss the smell of sweat I used to get. (P1, F, 39, 5m)

I am a teenager, it is important for me to smell good and look good, but at this time I think I smell heavy or burnt plastic. I literally feel disgusted with myself because I think my sweat smells bad. I ask my mother to smell me all the time, to change my clothes often. I have started to shower more often and use deodorant. (P10, M, 18, 9m)

I am a sportsman, I feel that I sweat but the smell isn't like my usual sweat smell. Sometimes I think my sweat smells like chips. I never used perfume before, but now I do. When I clean my dog's kennel I can't smell anything. (P11, M, 40, 9m)

I tell my wife and kids that they should absolutely tell me if I smell sweaty and I won't be offended. (P5, F, 49, 12m)

### 3.3 Threat to safety

This theme describes participants' experiences of safety threats and dangers due to loss of smell. Most of the participants reported that they burned food very often and were therefore concerned about consuming spoiled food. Participants also reported being concerned about their inability to sense signs of danger such as smoke or gas.

I work in the automation department, it bothers me that I can't recognize burning materials, this is a serious problem for my work…. (P7, M, 36, 9m)

I work at a natural gas worksite. I started to think that it would be dangerous not being able to smell a gas leak and actually it worries me. (P8, M, 42, 6m)

I started to burn food a lot because I couldn't smell. I had to throw away a lot of food, because I was worried that leftover food would poison me. (P5, F, 49, 12m)

### 3.4 Emotional responses

This theme describes the participants' experiences regarding the emotional problems that they experienced due to loss of smell and how they coped with them. In the study, participants reported that their social ties decreased due to the loss of the sense of smell (home, flowers, children) for a long time and that they missed the smell of their loved ones, nature and home. Some of the participants stated that they were diagnosed with anxiety and depression as a result of the problems that they experienced related to loss of smell. Subthemes were created based on these descriptions.

What makes me sad the most as a mother is not being able to smell my kid. (P4, F, 28, 5m)

… as I said I am a nurse. Stress of working during the pandemic, having COVID‐19 and then not being able to smell, they all affected me really negatively, I started taking antidepressants and isolating myself socially. I miss smelling. I want to eat shepherd salad and grilled chicken. Sometimes I see food I like in my dreams. I am really worried that I will never be able to smell again. (P6, F, 42, 11m)

After I got COVID‐19, my grandchild was born and I couldn't see him for a long time. And now even if I see him, I can't smell him. I would like to able to smell him. I miss the smell of my house and flowers. (P9, F, 56, 8m)

I'm worried that if the virus causes brain damage, my smell dysfunction will never recover. (P3, F, 38, 3m)

Convincing people that I can't smell anything was really hard for me. People told me to force myself to smell or to see a psychologist. Even the doctors didn't care much about my condition. (P1, F, 39, 5m)

I wish it was just the anosmia. Smelling spoiled/stinky things is much more disturbing. I wish I couldn't smell at all instead of smelling stink. I understand very well now that smelling is as precious to me as a limb. (P4, F, 28, 5m)

### 3.5 Coping with smell loss

This theme explores the participants' attempts to overcome their problems with smell loss and their experiences of the effects. Most of the participants reported that they tried to find solutions to their olfactory disorder on social media and the internet, and some of them sought medical help, sniffing rose, thyme, eucalyptus and lavender oil, using steroid nasal drops and sniffing coffee. However, they stated that their efforts were not completely successful.

I went to a doctor, I used cortisone tablets but it didn't work. (P6, F, 42, 11m)

I researched remedy on social media and the internet. Smelling coffee, orange peel, ozone therapy, nose drops… I decided to wait when I saw articles saying the sense of smell usually improves after 5‐6 months. (P8, M, 42, 6m)

The doctor recommended olfactory training with rose, thyme, eucalyptus, lavender oil; I used it for a while, but I thought it didn't work and gave up. Because I know they have strong odours, but not being able to smell even these disappointed me. (P9, F, 56, 8m).

When you eat, they tell you to eat with its flavor in mind, but unfortunately it doesn't work. Nothing works. (P3, F, 38, 3m)

## 4 DISCUSSION

Smell loss attracts less attention than other sensory losses and is usually not treated right away. However, if these symptoms persist, a crucial area that has to be considered is the psychological, emotional and social implications. This study investigated the experiences and coping methods of patients who experienced long‐term loss of smell after COVID‐19.^30^

The main points that stand out in the descriptive characteristics of the participants in the study can be summarized as follows: It was noteworthy that most of the participants interviewed in this study were female and young. In a retrospective study, it was shown that anosmia was associated with older age, female sex and race.^18^ In another study, it has been reported that among COVID‐19 patients, smell/taste loss is more common in young and female patients.^13^, ^31^ In contrast, in a study examining symptoms after COVID‐19, it was stated that no statistically significant difference was found between loss of taste and smell in terms of gender.^31^, ^32^ In general, women appear to utilize social media more often, which may lead to selection bias in a research such as this one. More research results are needed to confirm that women are more prone to loss of smell after COVID‐19 than men. Another noteworthy point is that the patients whom we interviewed also stated that their symptoms were mild enough to be treated for COVID‐19 at home. Smell loss in COVID‐19 may be associated with a milder clinical course.^33^ In a retrospective study, it is remarkable that anosmia was clearly low in terms of hospitalization, morbidity and mortality.^34^

Loss of smell can have a significant impact on a patient's quality of life, as it can affect their ability to detect smells that indicate danger, enjoy food and even recognize loved ones.^20^, ^35^ In this section, the areas where individuals participating in our research were affected by long‐term loss of smell after COVID‐19 are discussed under the main headings.

There is a close relationship between smell, taste and nutrition. Therefore, impairments in these senses significantly affect nutritional efficiency. In parosmia, one of the olfactory disorders, smells are perceived as distorted. Infections with germs or viruses, head trauma, neurological disorders and COVID‐19 are some of the causes. Parosmia is often transient; however, it can occasionally be persistent.^36^, ^37^ It is seen that participants with parosmia in our study could not mostly describe the smell of onion, garlic, pepper, lemon, meat, chicken and egg. They stated that they cannot exactly describe the smell of these things, but they used descriptors such as ‘rotten, spoilt, odd, like smell of carrion, sickening’. We even had participants who preferred not to smell at all rather than having impaired sense of smell due to the negative effect of parosmia. In a qualitative study,^15^ it was reported that as a result of the changed odour perception after COVID‐19, people perceived tastes such as soap, metallic, rotten, perfume and chemicals.

Although the mechanism of smell and taste loss in COVID‐19 disease has not been fully elucidated, there is an integral relationship between smell and taste.^11^, ^13^, ^30^ In the literature, it has been found that patients report that bacon and jam taste like soap, meat tastes like rotten meat and coke tastes like perfume and they mostly sense a metallic taste.^15^, ^36^ In our study, some participants who experienced ageusia (inability to taste) stated that they tasted sweet, sour, salty and bitter but could not detect their intensity and aroma. They also stated that they tend to add more salt and sugar because of the decrease in the intensity of the taste. Our participants reported that they switched to a diet that was mostly neutral in terms of smell, including high‐calorie carbohydrates and snacks, and therefore even gained weight. On the other hand, there were participants who reported losing weight due to loss of smell and taste. It has been reported in the literature that there are individuals who seek sensory satisfaction due to anosmia and taste disorder, similar to ours.^14^, ^15^, ^37^

It is reported that people with loss of smell are concerned about how their body odour is perceived.^22^ Because of this, the study participants stated that they took more showers, used more deodorant and perfume and even requested a close friend or relative to smell them. Also, it has been reported that, due to hygiene concerns, the establishment and maintenance of friendship of those who experience loss of smell are negatively affected.^22^ Anxiety about not being able to detect one's own bad body odour may cause one to give up social interactions with friends.

A healthy sense of smell enables avoidance of environmental hazards, especially those associated with fear and disgust. Moreover, the difficulty of detecting spoiled food can lead to consumption of spoiled food and even poisoning. These deficits in the sense of smell can negatively affect participants' safety.^38^ In our study, participants experienced food burning incidents and they faced some risks like not being able to smell spoilt food. In a retrospective study, it has been reported that patients with anosmia often have accidents like poisoning, gas leak and fire.^17^ In another study, an association was shown between an increase in the level of smell loss and experiencing a dangerous event.^18^ In another study of COVID‐19 patients, it was reported that 45% of the participants could not smell any smoke that others noticed. Fifty‐seven percent of the patients had experienced at least one dangerous incident and 36% had experienced at least two dangerous incidents. Environmental signals are also provided by smell in potentially lethal and hazardous circumstances. As a result, smell loss may lead to certain safety issues.^14^

During the COVID‐19 pandemic, most people experienced significant restrictions in social interaction and were unable to see family members or even close friends. Since communication at that time was mostly virtual, the importance of smell was greatly underestimated. As the pandemic abated, the increase in social interaction brought the problem to light. In our study, descriptions of longing related to the sense of smell, such as missing the smell of one's grandchild, a loved one, one's home, cleanliness and flowers, were frequently included. A systematic review of patients with loss of smell due to various aetiologies reported that episodes of depression after olfactory impairment occurred in 40%–76% of patients.^38^ Three participants in our study reported that they experienced sensory and emotional deprivation related to smell loss, that they were afraid that they may not be able to smell again and that they were diagnosed with anxiety and depression for various reasons such as sadness and social isolation. For this reason, the psychological, emotional and social effects of smell loss should not be neglected if it lasts for a long time because these effects can significantly affect people's self‐confidence and general well‐being.

Participants stated that they became worried when the smell loss was prolonged and some of them resorted to olfactory training based on the recommendation of doctors and social media. Five participants reported using olfactory training, and three participants reported using systemic and nasal corticosteroids in our study. The participants did not fully comply with the recommended prescription and stopped the treatment when they did not recover quickly. However, in the literature, olfactory training involving smelling aromatic scents (lemon, rose, clove and eucalyptus) twice a day, for 20 s each, for at least 3 months is recommended.^6^, ^7^, ^8^ Although corticosteroids are recommended in the literature, it is stated that the level of evidence on treatment efficacy is low.^7^, ^8^, ^9^, ^10^, ^11^, ^12^, ^13^, ^14^, ^15^, ^16^, ^17^, ^18^, ^19^, ^20^, ^21^, ^22^, ^23^, ^24^, ^25^, ^26^, ^27^, ^28^, ^29^, ^30^, ^31^, ^32^, ^33^, ^34^, ^35^, ^36^, ^37^, ^38^, ^39^

## 5 STRENGTHS AND LIMITATIONS

In the literature, it is stated that the sense of smell returns in approximately 1‐3 weeks after the diagnosis of COVID‐19^13^, ^14^, ^15^, ^16^, ^17^, ^18^, ^19^, ^20^, ^21^, ^22^, ^23^, ^24^, ^25^, ^26^, ^27^, ^28^, ^29^, ^30^, ^31^, ^32^, ^33^, ^34^, ^35^, ^36^, ^37^, ^38^, ^39^, ^40^ but there are limited data about the effects of long‐term smell loss. The strength of this study is that it adds valuable information to the small body of knowledge about the relevant experiences of participants with smell loss.

The diagnosis of smell loss of the participants in this study was based on their self‐reports, and an objective assessment of the severity of smell loss has not been performed. In addition, in the sections where the emotional and social effects of anosmia were discussed, the participants indicated that this situation did not cause any problems in their sex life and they refrained from talking about this topic. The question on participants' sex life, which was originally planned to be included, was not asked because data saturation was not achieved. The fact that the participants did not express opinions about how their sex life was affected may be due to the fact that sexuality is a difficult topic to talk about in our culture.

## 6 CONCLUSION

On the basis of this qualitative study, it was concluded that nutritional, safety, emotional, work and social lives of the individuals are adversely affected if the smell loss due to COVID‐19 lasts for a long time. Smell loss was left aside in the pandemic period because it is thought as an esthetical requirement should be carefully considered due to its long‐term negative effects after COVID‐19. Understanding and focusing on the negative effects of loss of smell may contribute to a solution to long‐term smell loss problems.

## AUTHOR CONTRIBUTIONS

**Hafize Özdemir Alkanat**: Conceptualization; methodology; writing—original draft; data curation; writing—review and editing; formal analysis; supervision; investigation; project administration. **Selda Arslan**: Conceptualization; methodology; writing—original draft; data curation; writing—review and editing; formal analysis.

## CONFLICT OF INTEREST STATEMENT

The authors declare no conflict of interest.

## ETHICS STATEMENT

Written informed consent was obtained from all participants.

## Data Availability

The data that support the findings of this study are available from the corresponding author upon reasonable request. The data are not publicly available due to ethical reasons. No additional data exist.
